# Surface-enhanced Raman spectroscopy method for classification of doxycycline hydrochloride and tylosin in duck meat using gold nanoparticles

**DOI:** 10.1016/j.psj.2021.101165

**Published:** 2021-03-27

**Authors:** Ting Wang, Muhua Liu, Shuanggen Huang, Haichao Yuan, Jinhui Zhao, Jian Chen

**Affiliations:** Key Laboratory of Modern Agricultural Equipment in Jiangxi Province, Jiangxi Agricultural University, Nanchang 330045, China

**Keywords:** duck meat, doxycycline hydrochloride, support vector machines, surface-enhanced Raman spectroscopy, tylosin

## Abstract

This paper investigated on 478 duck meat samples for the identification of 2 kinds of antibiotics, that is, doxycycline hydrochloride and tylosin, that were classified based on surface-enhanced Raman spectroscopy **(SERS)** combined with multivariate techniques. The optimal detection parameters, including the effects of the adsorption time, and 2 enhancement substrates (i.e., gold nanoparticles as well as gold nanoparticles and NaCl) on Raman intensities, were analyzed using single factor analysis method. The results showed that the optimal adsorption time between gold nanoparticles and analytes was 2 min, and the colloidal gold nanoparticles without NaCl as the active substrate were more conducive to enhance the Raman spectra signal. The SERS data were pretreated by using the method of adaptive iterative penalty least square method (air-PLS) and second derivative, and from which the feature vectors were extracted with the help of principal component analysis. The first four principal components scores were selected as the input values of support vector machines model. The overall classification accuracy of the test set was 100%. The experimental results showed that the combination of SERS and multivariate analysis could identify the residues of doxycycline hydrochloride and tylosin in duck meat quickly and sensitively.

## INTRODUCTION

With the development of social industrialization, the food safety has been paid close attention increasingly around the world. In recent years, antibiotics were widely used to prevent and treat diseases in animal husbandry. Using antibiotics as inhibitors of bacterial overgrowth and growth promoters is a common phenomenon in order to increase economic efficiency (Karacaglar et al., 2020). Macrolides antibiotics are produced by streptomyces and micromonospora, which have antibacterial and mycoplasma infection effects for their similar molecular structure, physical, chemical properties, and biological effects (Jansen et al., 2017). Macrolides are medium spectrum antibiotics, mainly including erythromycin, roxithromycin, kanamycin, tyrosine, etc. (Tenson et al., 2003; Yonath and Ada, 2005; Novak et al., 2009; Arsic et al., 2014). Tylosine **(TYL)**, a first-generation macrolides of 16 membered rings, is commonly used as special veterinary antibiotics to promote animal growth and treat mycoplasmas in farm animals (Devreese et al., 2012; Pinckney et al., 2016). Nevertheless, the presence of antibiotic residues may increase drug resistance of microbial strains in body, and produce allergic or toxic reactions for hypersensitive individuals, which pose a potential risk to human health (Wu et al., 2019). In addition, Doxycycline hyalite **(DCH)**, which also is called doxycycline hydrochloride, is a long-acting and broad-spectrum semi synthetic tetracycline antibiotic, and its molecular formula is C_22_H_26_N_2_O_9_. Due to its inexpensive cost, tetracycline antibiotics are usually used to prevent many diseases, promote the growth of animals and improve the effective production of poultry (Wang et al., 2018a). The use of DCH has greatly promoted the rapid development of breeding industry. At the same time, tetracycline antibiotics have relatively stable and a certain persistence. It leads to the residues of a part tetracycline antibiotic in the environment, which can indirectly or directly enter the body to pose a threat to public health (Samanidou et al., 2005; Lian et al., 2013). Therefore, the National Food Safety Standard on Maximum Residue Limits for Veterinary Drugs in Foods (GB31650-2019) in China has been issued to avoid excessive antibiotic residues in poultry, in which the permitted maximum residue limit of DCH and TYL in poultry was 100 μg /kg.

Many analytical technologies are used as detection methods in antibiotics. Among them, the most frequently used methods are liquid chromatography-tandem mass spectrometry, immunoassay and microbiological method etc. (Chafer-Pericas et al., 2011; Jank et al., 2017; Wu et al., 2020). Actually, although the above methods are mature and accurate, the pretreatment of samples is very demanding, and the detection process is time-consuming. Generally, they can only be used for sampling inspection, and are difficult to meet the requirements of rapid detection of antibiotic residues in meat specially in poultry. Consequently, it is great of research signification for a rapid, nondestructive, sensitive detection method of tetracycline and macrolide antibiotics.

Surface-enhanced Raman spectroscopy **(SERS)** is a sensitive analytical technique, of which the detection ability depends on the performance of SERS enhancement substrate due to the electromagnetic field near the rough precious metal surface, and its characteristics are simple preparation for samples, nondestructive and rapid data acquisition (Li et al., 2017; Wang et al., 2018b). Furthermore, silver and gold nanoparticles are the commonly used SERS enhancement substrates on account of having good performance and exciting local surface plasmons (Hassan, 2018). According to the status at domestic and international in recent years, Guo et al. used SERS technology to quickly detect the residues of oxytocin in duck meat by using gold nanoparticles and established a good linear relationship between the intensities of SERS signal at 1,271 cm^−1^ and the concentrations of duck meat extract containing oxytetracycline with coefficient of determination of 0.9891(Guo et al., 2017). Janči et al. developed a fast and sensitive method for detecting histamine in fish based on SERS, and the results indicated that the Partial least square **(PLS)** regression model based on spectral range 1139.9 to 1,644.7 cm^−1^ showed a linear trend with correlation coefficient predicted of 0.962 and ratio of performance to deviation of 7.250 (Janči et al., 2017). Cao et al. explored the characterization of the interactions between food-grade TiO_2_ nanoparticles and polymethoxyflavones using SERS (Cao et al., 2016). Zhou et al. carried out characterization analysis for food-grade TiO_2_ nanoparticles under the condition of simulated oral behavior, and SERS technology combined with PLS models was used to determine the blinding efficiency of mucin and TiO_2_ (Zhou et al., 2019). Gukowsky et al. established a rapid detection method of food colorants based on SERS, and the technology has been shown to be able to identify artificial and natural food colorants in the United States (Gukowsky et al., 2018). These studies showed that it would have a good ability to identify and classify antibiotic residues in duck meat if SERS was combined with multivariate analysis method. So far, there are few reports about the discriminant analysis of DCH and TYL by Raman spectroscopy coupled with support vector machines **(SVM)** modeling. Therefore, it is of great significance to investigate the screening methods of DCH and TYL in duck meat.

Herein, the aim of this paper was attempted to use SERS with the help of multivariate analysis to rapidly classify TYL and DCH residues in duck meat. To achieve this goal, the three pretreatments (i.e., adaptive iterative penalty least square (air-PLS, air-PLS and first derivative, as well as air-PLS and second derivative) were compared, and key principal components **(PCs)** were extracted as the inputs of SVM model to classify in the classification stage. This study provided a theoretical basis for the identification of multiple antibiotic residues in food with animal origin, and has potential application value in other fields, such as the differential diagnosis of non-hodgkin lymphoma in serum, and the identifying capsular of streptococcus pneumoniae isolates.

## MATERIALS AND METHODS

### Preparation of Samples

Preparation of 25 mM gold (III) chloride trihydrate (HAuCl_4_) solution: 1 g of solid HAuCl_4_ ·3H_2_O (not less than 49.0%, Sigma Aldrich Trading Co., Ltd., St. Louis, MO), was dissolved with 100 mL ultrapure water in a brown volumetric flask to obtain HAuCl_4_ solution (100 mg/L), from which 19.69 mL of HAuCl_4_ solution was taken out and put it into a 50 mL centrifuge tube. Next, 0.31 mL of ultrapure water was added to obtain 25 mM of HAuCl_4_ solution.

Preparation of TYL solution: 20 mg of TYL (about 98%, Germany Dr. Restorer Co., Ltd., Germany) was dissolved with 500 mL of ultrapure water in brown volumetric flask to obtain TYL solution (40 mg/L).

Preparation of DCH solution: 20 mg of DCH (about 98%, Nanchang Jingle Scientific Instrument Co., Ltd., China) was dissolved with 500 mL of ultrapure water in brown volumetric flask to obtain DCH solution (40 mg/L). The diverse concentrations of DCH solution, TYL solution, as well as DCH and TYL mixed solution were prepared using the above two standard solutions.

Preparation of duck meat samples: To start with, duck breast meat purchased from Hualian supermarket in Jiangxi Province was frozen in the refrigerator at −20°C for 5 h and sliced with a slicer. Next, the sliced duck meat was dried in a FD-1A-50Z vacuum freeze dryer (Beijing Toymaking Experimental Instrument Co., Ltd, China) for 24 h (Xu et al., 2020). Lastly, the duck meat samples were cut to about 0.3 × 0.3 cm and soaked into DCH solution, TYL solution or mixture solution of DCH and TYL to obtain samples containing DCH, samples containing TYL or samples containing DCH and TYL. All duck meat samples were divided into 4 groups, that is, 120 samples without DCH and TYL (control group), 119 samples containing DCH (DCH group), 119 samples containing TYL (TYL group), 120 samples containing DCH and TYL (DCH combined with TYL group).

### Synthesis of Gold Nanoparticles

The colloidal gold nanoparticles were synthesized according to a published protocol with slight modification (Bastus et al., 2011). In summary, 97.0 mg of solid trisodium citrate (analytical pure, Xilong Chemical Co., Ltd., Guangdong, China) was dissolved with 150 mL ultrapure water in a triangle round-bottomed flask and heated for 15 min until rolling boil with an intelligent constant temperature agitator. Then, 1 mL of 25 mM HAuCl_4_ solution was added drop-wise into the above sodium citrate solution under vigorous magnetic stirring. Subsequently, the reaction mixture was kept boiling under stirring for 10 min. In the meantime, the color change of the mixed solution could be observed from yellow to blue gray and to light pink. The colloidal gold nanoparticles were cooled to 90°C in a container, then immediately, 1 mL of 60 mM sodium citrate aqueous was added, and 1 mL of HAuCl_4_ solution also was added after waiting for 2 min. After 30 min at the same temperature, 2 mL of gold nanoparticles colloid were taken out. The above process was repeated, and the sizes of gold nanoparticles were constantly increased through 14 times of the repeated additions. Eventually, the colloidal gold nanoparticles were stored at the room temperature for further using.

### SERS Measurement

For measuring SERS spectra, about 50 μL of colloidal gold nanoparticles was dropped directly onto the duck meat sample on the glass slide, and then was illuminated after waiting for 2 min by a DXR micro Raman spectrometer (Thermo Fisher Scientific Co., Ltd., Waltham, MA) equipped with a semiconductor laser of 780 nm excitation wavelength fixed at 20 mW. In addition, 10 s preview acquisition time, 10 s sample exposure time and 16 s background exposure times were set. The SERS spectra in the wavenumber range of 50 to 3,000 cm^−1^ were used for data analysis.

The SERS spectrum of each sample was measured only one time. A total of 478 SERS spectra were measured for four groups of duck meat samples, of which two thirds (318) were randomly selected as the training set to establish the discriminant model, and the rest were used as the test set to verify the classification accuracy. The validity of the discriminant model was verified by the classification accuracy (i.e., the ratio of the number of samples correctly predicted by the model to the total number of samples).

### Optimization Schemes of SERS Detection Parameters

SERS enhancement substrates and absorption time are the important factors affecting on SERS intensities. Accordingly, the effects of 3 SERS detection parameters (i.e., 2 enhancement substrates, addition of colloidal gold nanoparticles and absorption time) on SERS signal intensities were investigated using the single factor test method. Five parallel samples were performed for the optimization of SERS detection parameters, and the average values of their spectra were taken as the SERS spectrum of the sample. The optimum detection parameters were determined by comparing the SERS signal intensities at 558 and 594 cm^−1^.

In order to investigate the effect of adsorption time on SERS intensities, 50 μL of colloidal gold nanoparticles was added onto the duck meat samples. Then, after 0, 2, 4, 6, and 8 min of adsorption times, the spectra were measured, respectively.

In order to investigate the effect of SERS enhancement substrates on SERS intensities, 50 μL of 2 SERS enhancement substrates (i.e., colloidal gold nanoparticles, and colloidal gold nanoparticles mixed with 100 mg/L NaCl solution) was added onto the duck meat samples, respectively. After 2 min of adsorption time, their SERS spectra were measured.

### Data Analysis

All data analyses were performed using the Unscrambler X 10.4 and MATLAB 2014a softwares. Three pretreatment methods, namely, air-PLS, air-PLS and first derivatives as well as air-PLS and second derivatives, were compared to select the optimal pretreatment. PCA algorithm was used to extract the features of SERS data. In addition, the first 4 PCs scores were used as input values of SVM model to realize the aim of 4 groups samples classification.

## RESULTS AND DISCUSSION

### SERS Spectra Analysis of Samples

**Figure 1:**
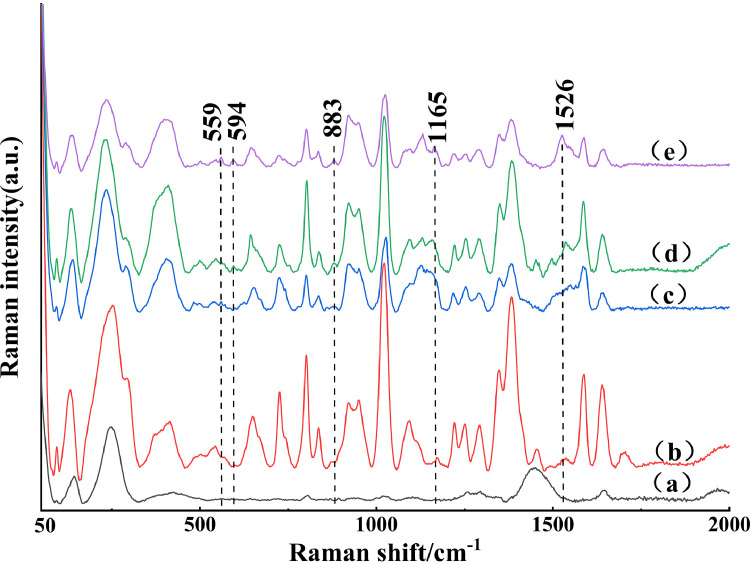
Representative SERS spectra of (a) background of gold nanoparticles (b) control group, (c) DCH group, (d) TYL group, and (e) DCH combined with TYL group. Abbreviations: DCH, doxycycline hydrochloride; TYL, tylosin.

The average SERS spectra of background of gold nanoparticles and 4 groups, that is, control group, DCH group, TYL group and DCH combined with group, were displayed in Figure 1. Obviously, the Raman peaks could be observed at 559, 594, 883, 1,165 and 1,526 cm^–1^ from curve (e), which didn't present on the spectra of control group and background of gold nanoparticles. Therefore, they could be selected as Raman peaks to distinguish whether there were DCH or TYL residues in duck meat. Nevertheless, the four groups couldn't be distinguished entirely in these Raman peaks. So further analyzing of curve (c) and (d), 559 and 594 cm^−1^ could be considered as the SERS feature peaks to identify DCH and TYL residues in duck meat.

**Figure 2:**
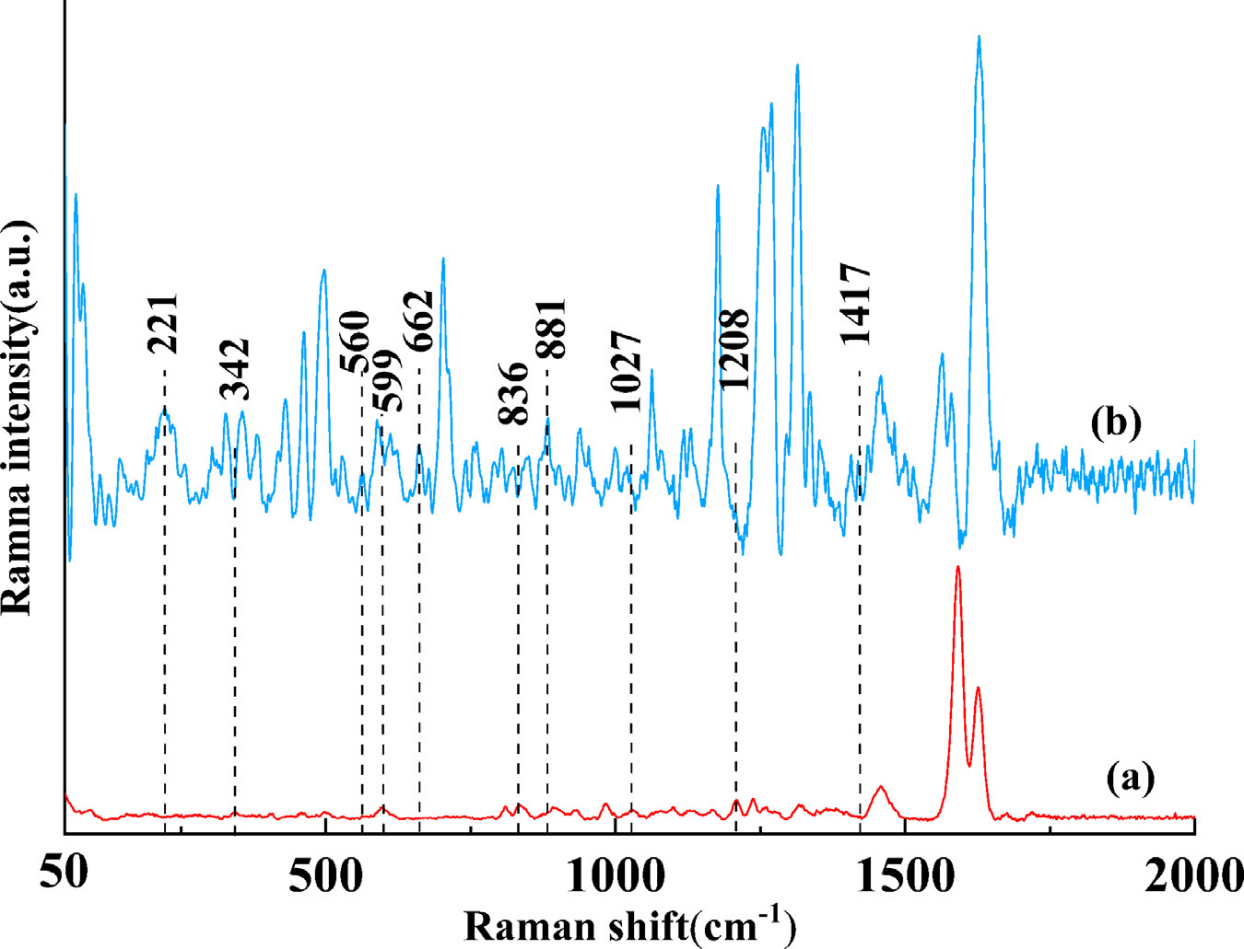
The SERS spectra of solid TYL (a) and solid DCH (b). Abbreviations: DCH, doxycycline hydrochloride; SERS, surface-enhanced Raman spectroscopy; TYL, tylosin.

To verify whether the above peaks were the feature peaks of DCH and TYL in duck meat, the solid DCH and solid TYL were used to analyze the spectral characteristics. It was evident that the prominent Raman peaks at 342, 599, 836, 1,027 and 1,208 cm^−1^ could be observed simultaneously from curve (a) in Figure 2 and didn't overlap with solid DCH from curve (b). Namely, they were the characteristics peaks of solid TYL. Similarly, by comparing whether the characteristic peaks on curve (b) were overlapped with those on curve (a), it could be concluded that 221, 560, 662, 881, and 1,417 cm^−1^ were the characteristic peaks of solid DCH.

By the comprehensively analysis of SERS characteristic peaks of solid DCH, solid TYL as well as DCH and TYL in duck meat, the results showed that the characteristic peaks of solid DCH and solid TYL were closed to those of DCH and TYL in duck meat at 559 and 594 cm^−1^. It maybe owing to the influence of protein and fat in duck meat itself so that the Raman spectra characteristic peaks of DCH and TYL in duck meat had been blue shifted by 1 and 5 nm, respectively. On the whole, the characteristic peak at 559 cm^−1^ could be used to determine whether there was DCH residues in duck meat, and 594 cm^−1^ could be used to estimate whether there was TYL residues in duck meat.

### Optimization of SERS Detection Parameters

**Figure 3:**
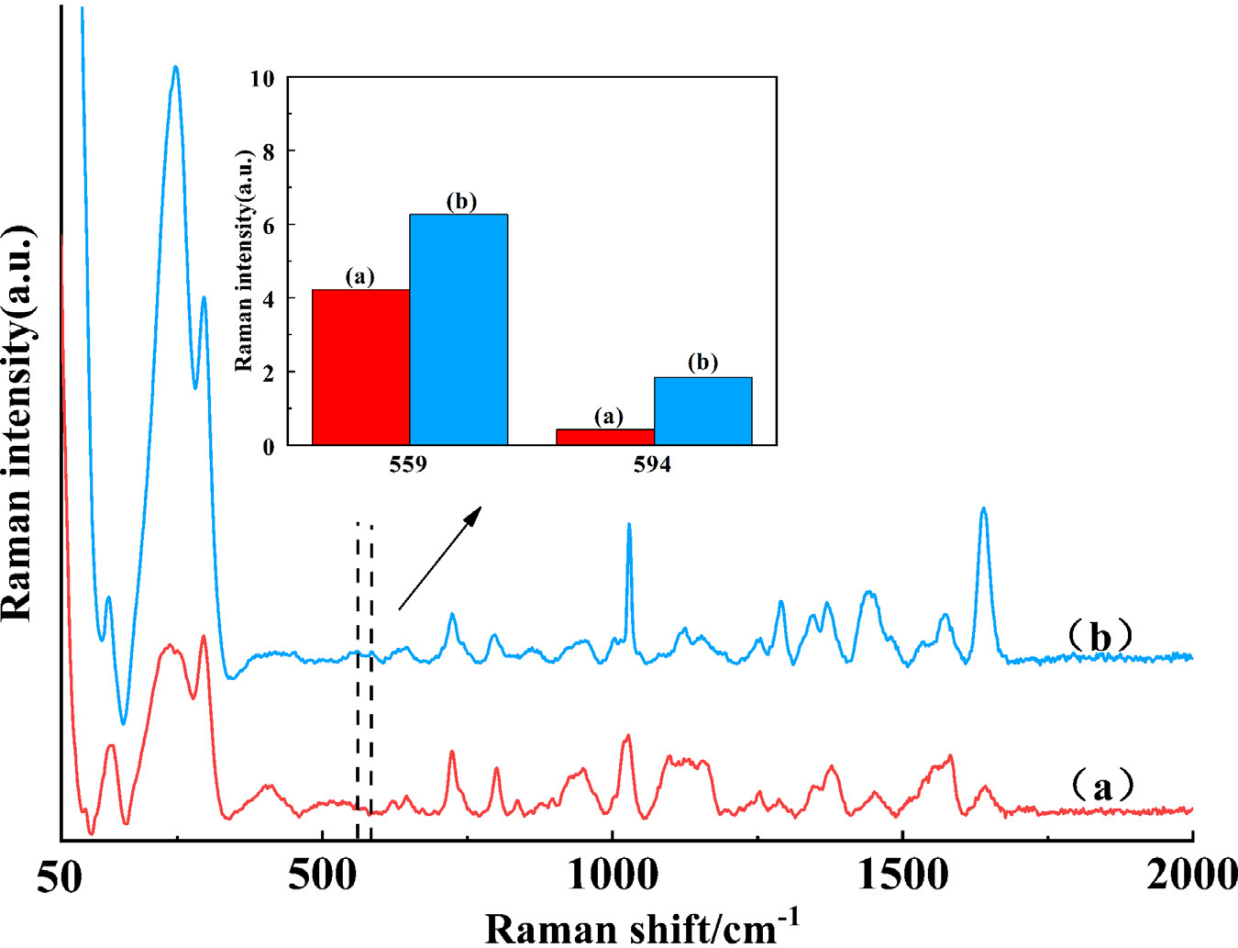
The SERS spectra of samples for 50 μL gold nanoparticles mixed with 100 μL NaCl solution (curve a) and 50 μL of gold nanoparticles (curve b) were respectively added onto samples of DCH combined with TYL group. The inset was the mean Raman intensity at 559 and 594 cm^−1^ corresponding to curves (a) and (b). Abbreviations: SERS, surface-enhanced Raman spectroscopy.

When the gold nanoparticles meet with the standard solution molecules, the molecules were gathered in the gap of the gold nanoparticles rough surface with adsorption capacity to enhance the SERS spectra (Battocchio et al., 2014). At this time, if some electrolytes, such as NaCl and magnesium sulfate, are selected as one part of enhancement substrate, it may further enhance or reduce the Raman spectral intensities (Tang et al., 2013; Luan et al., 2015).Therefore, we analyzed the enhancement effect of 2 enhancement substrates (i.e., gold nanoparticles, gold nanoparticles and NaCl) on the samples of DCH combined with TYL group, and their spectra were shown in Figure 3. Through observation, the spectrum of curve (a) was smaller than that of curve (b) on the whole, and the histogram in the illustration could highlight that the gold nanoparticles substrate could stimulate the SERS signals of DCH and TYL in the duck meat more than the gold nanoparticles + NaCl. It has been shown that adding Cl^−^ could promote the aggregation of metal colloids, generate a large number of SERS hotspots in the gap between particles, and produce a significant enhancement effect. However, the enhancement effect of SERS signal could also be inhibited in the condition of a great quantity of Cl^−^ (Pong et al., 2007; Pamela, 2017). With the increase of Cl^−^ content, the ratio of surface gold nanoparticles to analyte were decreased, which the SERS signal could be abated (Gong et al., 2019). When NaCl solution was added as an active agent, the Raman characteristic peak intensities of samples at 559 and 594 cm^−1^ would become more weaken. So, only gold nanoparticles were chosen as SERS enhancement substrate in the subsequent experiments.

**Figure 4:**
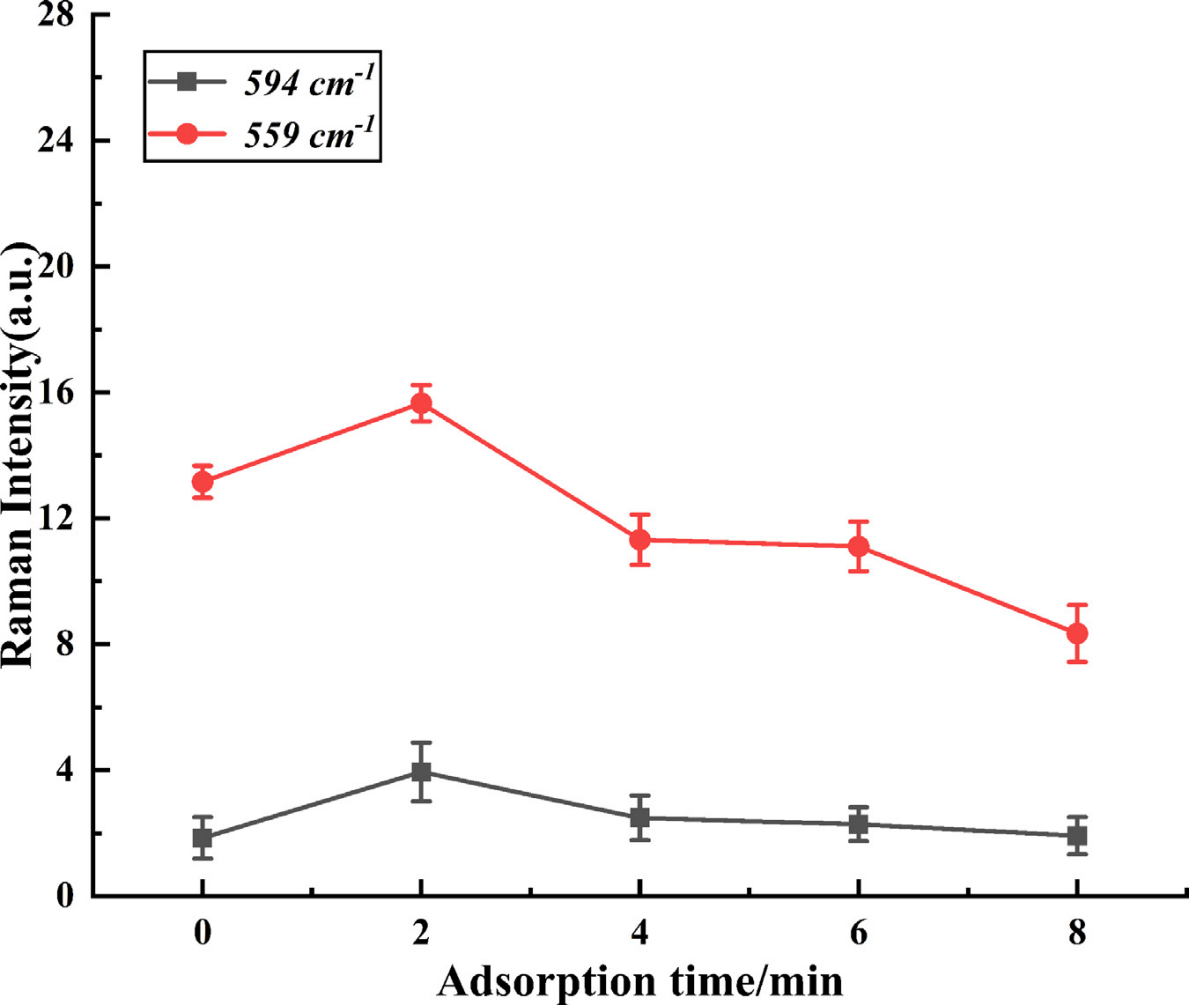
The effect of the different absorption times on SERS signal intensities. Abbreviations: SERS, surface-enhanced Raman spectroscopy.

When the analyte was adsorbed on the surface of gold nanoparticles, its adsorption time has an important effect on enhancing SERS signal intensities (Evelyn et al., 2015). While the analyte contacts with the enhanced substrate, the active “hot spot” would be generated at a certain moment to make the SERS signal reach the maximum. As shown in Figure 4, the influence of the different adsorption times on SERS signal intensities at 559 and 594 cm^−1^. With the increases of the adsorption times, the intensity of Raman spectra reached the maximum in 2 min, and then decreased gradually at 559 cm^−1^.The intensities of SERS signal became higher at the initial stage, it may be that the availability of more than required number of active hot spot on the surface, and the intensities of SERS spectra became slower at the later stages of contact time due to lesser number of active hot spot(Pal and Deb, 2014). Besides, it has no obvious tendency at 594 cm^−1^, and the maximum of Raman intensities were at 2 min. Therefore, 2 min was selected as the optimal adsorption time.

### Data Dimension Reduction and Classification

Spectral pretreatment is an effective method to dispose spectral disturbance noise and concurrently retain the information of chemometrics analysis (Albanell et al., 2012; Balabin and Smirnov, 2012). Data pretreatment plays a very important role in the analysis of near-infrared, Raman and other spectra. Because of the disturbance of background noise or instrument baseline drift in the process of spectral acquisition, spectral data inevitably contains some useless information. Therefore, in order to reduce the unnecessary information in the data to improve the classification accuracy of the samples, multifarious chemometrics algorithms have been put forward for spectral pretreatment (Chen et al., 2019). In present study, it compared the effects of 3 pretreatment methods, that is, air-PLS, air-PLS and first derivative, as well as air-PLS and second derivative, on the classification of four groups samples (n = 478). Table 1 displayed the numbers of correct and erroneous after each group was processed by three pretreatment methods combined with PCA-SVM for test set (n = 160). The classification accuracies of the PCA-SVM model for training set were respectively 82.7%, 100%, and 100% under these 3 pretreatment methods, and their classification accuracies for test set were 77.5%, 96.2%, and 100%, respectively. To put it simply, air-PLS and second derivative was selected as the optimal pretreatment method.

**Table 1:** Results of classification of DCH and TYL in duck meat based on PCA-SVM with three pretreatment methods for test set.

		Classified as			
Pretreatmet methods	Groups	Control	DCH	TYL	DCH combined with TYL
Air-PLS	Control	40	0	0	0
	DCH	0	36	0	6
	TYL	0	1	26	13
	DCH combind with TYL	0	16	0	24
Air-PLS and first derivative	Control	40	0	0	0
	DCH	0	39	1	0
	TYL	0	4	35	1
	DCH combind with TYL	0	0	0	40
Air-PLS and second derivative	Control	40	0	0	0
	DCH	0	40	0	0
	TYL	0	0	40	0
	DCH combind with TYL	0	0	0	40

Abbreviations: DCH, doxycycline hydrochloride; PLS, Partial least square; SERS, surface-enhanced Raman spectroscopy; TYL, tylosin.

**Figure 5:**
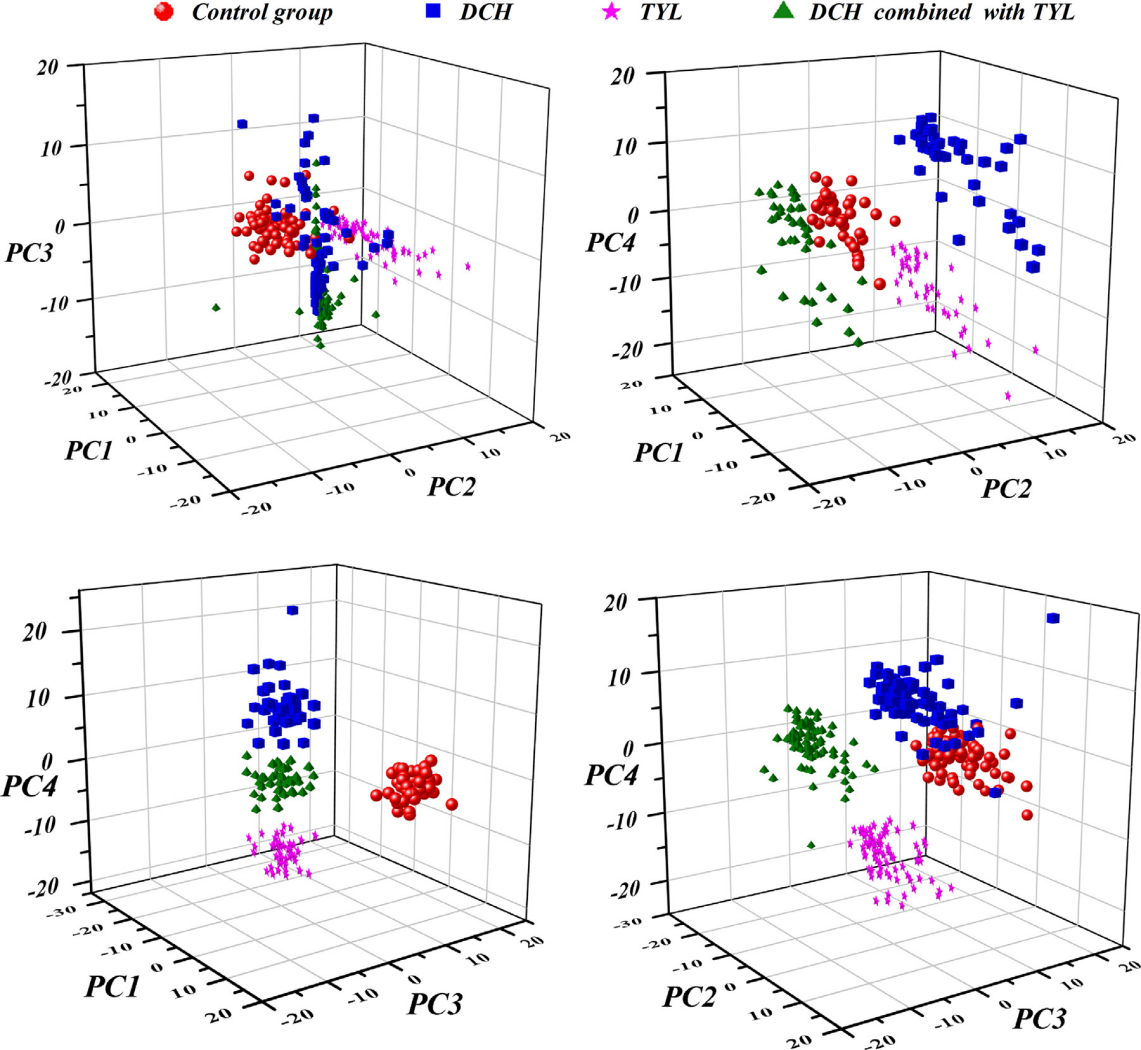
3-D scatter plots after training data dimension reduction, constructed by selected first four PCs scores calculated from PCA. Abbreviations: PCs, principal components; PCA, principal component analysis.

PCA, a kind of linear and unsupervised visualization technology, is often used to extract the main features of the information from data with less message loss after dimensionality reduction (Khulal et al., 2016). It is primarily the reconstruction of the feature space of the original spectral data through orthogonal transformation, transforming the correlation information into linear uncorrelated variable, that is, PCs (Krähmer et al., 2015). To visualize the score results of PCA, the first 4 PCs scores were used to construct 3-D scatter plots as shown in Figure 5. Where, 4 groups of samples were observed in the space of PC1-PC2-PC3 and they couldn't be distinguished owing to the overlapping. In this case, the selection of PCs should be further considered. By analyzing the space of PC1-PC2-PC4, the DCH group was mainly distributed in the central area and could be distinguished from the other 3 groups. Moreover, DCH group, TYL group and DCH combined with TYL group occupies a small part of their respective space in the space of PC1-PC3-PC4, however, the remaining control group was gathered in different space areas and could be identified. DCH combined with TYL group could be completely separated from other groups in PC2-PC3-PC4. In summary, the four groups of samples could be distinguished completely under the condition of no less than the first 4 PCs. Therefore, the first 4 PCs scores were selected as the input values of the classification model.

SVM, which is a kind of modeling method that can identify, classify samples and do regression analysis is also a new generation of machine learning algorithm based on statistical learning theory, and is mainly embodied in dealing with the problem of nonlinear indivisibility (Guo et al., 2018). It is necessary to adhibit a kernel function at such a time. That means, the samples are mapped from the original space to the feature space, and in which it can be divided linearly, so as to solve the problem of nonlinear classification. Therefore, the choice of kernel function has a great influence on SVM. In this experiment, the first four PCs scores were used as the input values of SVM classification model, and C-SVM was used as the type of SVM model to realize the multivariable SVM classification method. The efficiency of radial basis function kernel was stronger than polynomial kernel in the nonlinear analysis determined by arithmetic of the cross-validation, so the radial basis function was used as the kernel function of classification model (Cho et al., 2016). Furthermore, the classification performance of SVM model was tightly bound to the penalty parameter C and the kernel parameter g. If the penalty parameter was too large or incline to zero, this would lead to SVM over-fitting or under-fitting, resulting the error of classification. Therefore, it was indispensable to optimize them automatically, and the optimal values of these two parameters C and g were 0.01 and 1, respectively. SVM classification model combined with 3 pretreatment methods to analyze the sensitivity and specificity values of each groups for the test set just as shown in Table 2. Here, by using the pretreatment method of air-PLS and second derivative, the sensitivities and the specificities of samples of each group were all 100% and 100%, respectively. These results indicated that the adopted SERS method with the help of SVM model was a stable and reliable method for the identification of DCH and TYL residues in duck meat. This was of great significance for the rapid identification of antibiotic residues in poultry for food safety detection.

**Table 2:** Sensitivity and specificity values of PCA-SVM model with different pretreatment methods for classification of DCH and TYL in duck meat for test set.

	Air-PLS		Air-PLS and firstderivative		Air-PLS and secondderivative	
Groups	Sensitivity	Specificity	Sensitivity	Specificity	Sensitivity	Specificity
Control	100%	100%	100%	100%	100%	100%
DCH	90%	81%	98%	97%	100%	100%
TYL	65%	100%	88%	99%	100%	100%
DCH combind with TYL	60%	81%	100%	99%	100%	100%

Abbreviations: DCH, doxycycline hydrochloride; PCA, principal component analysis; SVM, support vector machines; SERS, surface-enhanced Raman spectroscopy; TYL, tylosin.

## CONCLUSIONS

To sum up, this study emphasized a simple, rapid and sensitive method based on SERS coupled with multivariate data analysis, which could become an effective antibiotic identification and analysis technology to classify DCH and TYL residues in duck meat. In the process of experimental investigation, the influences of 3optimized parameters (i.e., the adsorption time and two enhancement substrates) on SERS spectral intensities were considered. The results showed that the SERS spectra of samples had the better enhancement effects under the condition of only gold nanoparticles without NaCl as enhancement substrate and 2 min of the adsorption time. In order to improve the classification accuracy of the model, the three pretreatment methods were compared, and air-PLS and second derivative were selected as the optimal pretreatment method. The four groups of training set samples could be wholly discriminated by using the first four PCs scores as the input values of SVM model. The SVM classification model was verified using the test set to achieve 100% classification accuracy. Therefore, the innovative method of using SERS technology with the help of multivariate statistical discriminant model could meet the requirements of rapid identification of DCH and TYL residues in duck meat. The identification of multidrug residues in animal meat can be explored on the basis of this method, expanding the detection technology field in further.
